# Leveraging PARP-1/2 to Target Distant Metastasis

**DOI:** 10.3390/ijms25169032

**Published:** 2024-08-20

**Authors:** Mallory I. Frederick, Djihane Abdesselam, Anna Clouvel, Laurent Croteau, Saima Hassan

**Affiliations:** 1 Faculty of Medicine, Université de Montréal, Montreal, QC H3C 3T5, Canada; mallory.frederick@umontreal.ca (M.I.F.); djihane.abdesselam@umontreal.ca (D.A.); laurent.croteau@umontreal.ca (L.C.); 2 Centre de Recherche du Centre Hospitalier de l’Université de Montréal (CRCHUM), l’Institut de Cancer de Montreal, Montreal, QC H2X 0A9, Canada; anna.clouvel@gmail.com; 3 Division of Surgical Oncology, Department of Surgery, Centre Hospitalier de l’Université de Montréal (CHUM), Montreal, QC H2X 0C1, Canada

**Keywords:** PARP-1, PARP-2, PARP inhibitors, cancer, tumor microenvironment, metastasis

## Abstract

Poly (ADP-Ribose) Polymerase (PARP) inhibitors have changed the outcomes and therapeutic strategy for several cancer types. As a targeted therapeutic mainly for patients with *BRCA1/2* mutations, PARP inhibitors have commonly been exploited for their capacity to prevent DNA repair. In this review, we discuss the multifaceted roles of PARP-1 and PARP-2 beyond DNA repair, including the impact of PARP-1 on chemokine signalling, immune modulation, and transcriptional regulation of gene expression, particularly in the contexts of angiogenesis and epithelial-to-mesenchymal transition (EMT). We evaluate the pre-clinical role of PARP inhibitors, either as single-agent or combination therapies, to block the metastatic process. Efficacy of PARP inhibitors was demonstrated via DNA repair-dependent and independent mechanisms, including DNA damage, cell migration, invasion, initial colonization at the metastatic site, osteoclastogenesis, and micrometastasis formation. Finally, we summarize the recent clinical advancements of PARP inhibitors in the prevention and progression of distant metastases, with a particular focus on specific metastatic sites and PARP-1 selective inhibitors. Overall, PARP inhibitors have demonstrated great potential in inhibiting the metastatic process, pointing the way for greater use in early cancer settings.

## 1. Introduction

The vast majority of metastatic cancer patients still die from cancer [1]. The 5-year relative survival of patients with metastatic cancers, such as breast, prostate, ovarian, or pancreatic cancers, remains alarmingly low, at less than 37% [2,3,4,5]. Nonetheless, targeted therapeutic agents are emerging as a gamechanger in the treatment paradigm for cancer patients by prolonging survival with less toxicity than standard chemotherapy [6]. One such family of therapeutic agents that improved the overall survival of patients with metastatic disease and early cancers is that of Poly (ADP-Ribose) Polymerase inhibitors (PARPi) [7,8].

PARPi mainly target PARP-1/2, enzymes that belong to a larger 17-member PARP family [9]. PARP proteins are commonly localized in the nucleus but can also be found in the cytoplasm or cell membrane. Most members of the PARP family catalyze the transfer of ADP-ribose polymers onto proteins, in a process called poly(ADP-ribosyl)ation or PARylation [10]. PARP proteins help maintain cellular homeostasis by participating in various biological processes, including DNA repair and cell cycle regulation (PARP-1/2/3); chromosome structure (PARP-1/2); transcription (PARP-1/2/7/10/14); inflammation (PARP-1/2/5a/5b/14); metabolic regulation (PARP-1/2/5a/5b/14); and RNA processing (PARP-1/5a/7/10/12/13/14/15) [9]. PARPi have two main mechanisms of action: synthetic lethality and PARP-DNA trapping [11]. The synthetic lethal interaction of PARPi and BRCA1/2 was one of the earlier described mechanisms in which PARPi led to single-strand breaks, followed by double-strand breaks, resulting in genomic instability and, ultimately, cell death. In PARP-DNA trapping, PARP-1 cannot be released from the site of DNA damage, thereby forming a cytotoxic lesion with ensuing cell death. PARPi differ in their PARP-DNA trapping potency, and four of the most potent PARPi, namely, talazoparib, niraparib, olaparib, and rucaparib, have received approval by the U.S. Food and Drug Administration (FDA) [12].

Orally available, PARPi are currently used in several cancer types, including breast, prostate, ovarian, and pancreatic cancers [7]. One of the most aggressive subtypes of breast cancer is triple-negative breast cancer (TNBC), which lacks overexpression of estrogen and progesterone receptors and human epidermal growth factor receptor 2 (HER2). Approximately 11–20% of TNBCs have a germline mutation in *BRCA1* [13]. These patients benefit from PARPi either in the metastatic or adjuvant settings (after completion of initial treatments upon diagnosis) [14,15]. Second, amongst prostate cancer patients, metastatic castrate-resistant prostate cancer (CRPC) is resistant to androgen deprivation therapy and considered incurable [16]. PARPi are offered to metastatic CRPC patients with somatic or germline mutations in *BRCA1/2* (gBRCA^MUT^), or homologous recombination-deficient (HRD) tumors, either in first-line combination therapy or second-line monotherapy [17]. Third, in ovarian cancer, PARPi are used in patients with somatic or gBRCA^MUT^, HRD tumors, or unselected patients as maintenance therapy after completion of therapy, either for recurrent disease or at primary presentation [18]. Finally, gBRCA^MUT^ patients with metastatic pancreatic cancers can benefit from PARPi as maintenance therapy [19]. Overall, the use of PARPi is expanding beyond gBRCA^MUT^ patients, including HRD tumors and unselected patients.

The current landscape of clinical trials with PARPi is evaluating approaches to overcome tumor resistance and toxicity with new combination approaches and the use of next-generation PARPi that are selective to PARP-1 [7,20]. One of the challenges in the clinical implementation of PARPi stems from limitations in pre-clinical studies. Much of the pre-clinical literature has focused on the anti-proliferative effect of PARPi, induction of DNA damage, and inhibition of primary tumor growth in mouse models [21,22,23]. While PARPi efficacy was shown in both BRCA^MUT^ and *BRCA1/2*-wild type (BRCA^WT^) models, the impact of PARPi on the initiation or progression of distant metastasis is not well understood. While only a limited number of studies have assessed the impact of PARPi on various components of the metastatic process, such as angiogenesis and epithelial-to-mesenchymal transition (EMT) [24,25], there is a growing body of literature evaluating PARPi in combination, providing an improved understanding of the mechanisms at the metastatic sites [26,27].

To better understand the potential of PARPi, we present a comprehensive review of PARP-1 biology—the nuclear and extracellular functions of PARP-1; the impact of PARP-1 in cancer progression, with particular attention to the tumor microenvironment and distant metastasis; the preclinical efficacy of PARPi in impeding metastasis; and finally, the clinical advancements of PARPi in the early-cancer and metastatic contexts.

## 2. PARP-1 Structure, Regulation, Nuclear Functions, and Role in Inflammatory Response

### 2.1. PARP-1 Structure, Regulation

#### 2.1.1. PARP-1 Structure

PARP-1 is a 113 kDa protein with six domains. PARP-1 consists of five DNA-binding domains, including three zinc-finger (ZnFI, ZnFII, ZnFIII) domains, an automodification or *BRCA1* C-terminus (BRCT) domain, the tryptophan-glycine-arginine-rich WGR domain, and the C-terminus catalytic (CAT) domain, which consists of the helical domain (HD) and ART domains [28,29,30]. ZnF1 and ZnFII are required for binding to DNA damage sites. ZnFIII is implicated in the DNA-dependent enzymatic activity of PARP-1. The BRCT domain regulates protein–protein interactions and is involved in PARP-1 homodimerization and heterodimerization, particularly with other DNA repair proteins that also contain a BRCT domain. The WGR domain interacts with DNA, alongside ZnF1 and ZnFII. The HD acts as an inhibitor of the ART domain. The ART domain includes amino acids involved in the catalysis and binding of NAD+. Therefore, in the absence of DNA damage, the folded HD blocks the access of NAD+ to the ART domain, keeping PARP-1 at a minimal basal activity level [31].

Upon interaction with DNA containing double-strand breaks, PARP-1 undergoes a change in structural conformation, in which the PARP-1/DNA complex becomes more compact [32]. Herein, the WGR domain interacts with the ZnFI and ZnFIII domains, forming a DNA-binding interface. This reconfiguration causes a molecular switch in the HD, modifying the flexibility and dynamics of the ART domain, resulting in significant PARP-1 activity. The automodification domain also becomes situated near the active site, increasing the propensity for PARP-1 to PARylate itself [28,32].

#### 2.1.2. PARP-1 Regulation

PARP-1 is a critical sensor of DNA damage. One of the earliest events in the DNA damage response (DDR) is the recruitment of PARP-1 to the site of DNA damage, serving to recruit other DNA repair proteins [33]. The function of PARP-1 in the DDR relies heavily on post-translational modifications of PARP-1 including PARylation, phosphorylation, sumoylation, acetylation, and ubiquitylation [34]. PARP-1 is responsible for approximately 85% of total PAR synthesis after DNA damage [35]. Following DNA damage, PARP-1 is stimulated by histone PARylation factor 1 (HPF1), leading to the auto-PARylation and activation of PARP-1 [36]. PARP-1 phosphorylation by ERK1/2 was also shown to be required for maximal PARP-1 activation in the setting of DNA damage [37]. PARP-1 acetylation was first demonstrated in the context of nuclear factor (NF)-κB-dependent transcription in immune cells [34]. As a result of stress responses, PARP-1 acetylation leads to PARP-1 activation independent of DNA damage in cardiomyocytes. Sumoylation affects the transcriptional activity of PARP-1, decreasing the expression of PARP-1-regulated target genes [38]. Ubiquitylation of PARP-1 leads to PARP-1 degradation [34]. In the context of PARP-DNA trapping, sumoylation followed by ubiquitylation of PARP-1 leads to the removal of trapped PARP-1 from chromatin [39].

### 2.2. Nuclear Functions of PARP-1

#### 2.2.1. DNA Repair and Maintenance of Genomic Integrity

PARP-1 is one of the key players in repairing DNA damage and maintaining genomic integrity [34]. Indeed, the depletion of PARP-1 in embryonic fibroblasts leads to DNA repair defects and chromosomal abnormalities. PARP-1 participates in various DNA repair pathways, including base excision repair (BER), single-strand break (SSB) repair, and double-strand break (DSB) repair. PARP-1 promotes BER by binding to SSB intermediates and by rapid recruitment of XRCC1, POLB, and LIG3 [40]. In SSB repair, PARP-1 rapidly binds to SSBs and recruits the scaffolding protein XRCC1, which aids in end processing by PNKP and aprataxin. POLB and LIG3 then complete the repair with DNA synthesis and ligation. In response to DSBs, PARP-1 contributes to both homologous recombination (in S and G2-phases of cell cycle), and classical non-homologous end-joining (c-NHEJ) (in all phases of cell cycle). In the context of homologous recombination, PARP-1 facilitates the recruitment of MRE11, NBN, and BRCA1, which allow 5′-end resection. PARP-1 also interacts and PARylates DNA-PKcs, which is instrumental in guiding the downstream events of the c-NHEJ pathway.

In addition to DNA repair, PARP-1 is also involved in the cellular response to replicative stress [41]. PARP-1 is recruited to stabilize the stalled replication fork which prevents its collapse. Once the fork is stabilized, PARP-1 participates in restarting replication by different mechanisms such as fork reversal. Moreover, the role of PARP-1 in regulating fork speed was recently demonstrated in cancer cells [42]. We and others have demonstrated that PARPi was associated with an increase in replication fork speed and DNA damage [27,43].

#### 2.2.2. PARP-1 Role in Transcriptional Regulation

PARP-1 controls transcription through four main mechanisms [44,45,46]. First, PARP-1 can modify chromatin accessibility. This can be achieved by altering chromatin structure through binding of PARP-1 to nucleosomes, modifying histone proteins, or regulating chromatin composition. Second, PARP-1 can function as an enhancer-binding factor by binding to specific DNA sequences or structures. Third, PARP-1 acts as a co-regulator of transcription factors. Here, the promoter-specific exchange of factors can occur, whereby inhibitory factors are released and stimulating factors are recruited. Fourth, by PARylating histones and other chromatin-associated proteins, PARP-1 also functions as an insulator, by restricting enhancer effects on promoters or preventing heterochromatin spread.

### 2.3. Inflammatory Responses of PARP-1

PARP-1 is implicated in maintaining the expression of cytokines, chemokines, and other inflammatory mediators [47]. These include tumor necrosis factor (TNF)-α, interleukin (IL)-1/6, and interferon (IFN)-γ. PARP-1 increases the expression of adhesion molecules such as vascular cell adhesion molecule, P-selectin, and E-selectin. Furthermore, chemoattractant chemokines are also upregulated with PARP-1, namely IL-8, macrophage inflammatory proteins (MIP)-1/2, including MIP-1α (also known as C-C motif chemokine ligand 3 (CCL3)), and monocyte chemoattractant protein-1 (MCP-1) or CCL2. As a result, PARP-1 knockdown or inhibition commonly leads to decreased expression of such chemokines [48,49,50,51,52,53,54], thereby inhibiting cell migration to inflammatory sites [47]. In particular, the modulation of CCL2 by PARP-1 was established in several pathophysiological contexts [48,49,50,51,52]. Importantly, an 18-fold reduction in CCL2 levels was identified in PARP-1 knockout mice in the setting of infection [51]. PARP-1 promoted the migration of natural killer (NK) cells to the site of infection, via NF-κB-mediated production of CCL2 by macrophages. One chemoattractant chemokine, stromal cell-derived factor-1 (SDF-1), is known to induce the migration of hematopoietic progenitor cells, endothelial cells, and leukocytes, mainly through interaction with its receptor, CXCR4 [55]. PARP-1 negatively regulates SDF-1 expression [56,57]. PARP-1-deficient cells were associated with a pronounced demethylation at the SDF-1 promoter, thereby increasing expression of SDF-1. PARP-1 can also bind directly to the SDF-1 promoter during the early stages of oxidative stress, which in turn leads to SDF-1 downregulation.

PARP-1 has also been shown to mediate inflammatory responses in different contexts. Inhibition of phagocytosis and clearance of apoptotic cells is mediated by high-mobility group box 1 (HMGB1), which promotes chemotaxis and accumulation of neutrophils to inflammatory sites [58]. Once PARylated by PARP-1, HMGB1’s ability to inhibit the clearance of apoptotic cells is enhanced, thereby promoting inflammation. In vivo, PARP-1 knockout mice lose the ability to recruit inflammatory factors in response to myocardial ischemia [59]. A similar effect was seen in neuronal damage, where microglial migration was dependent on PARP-1 expression [60]. Inhibition of the inflammatory response by the PARPi veliparib improved outcomes following intracerebral hemorrhage in mice [61]. Such a role for PARP-1 in regulating the inflammatory response was recently linked with ubiquitination and subsequent degradation of PARP-1 by RNF146 [62]. Interestingly, the production of several inflammatory cytokines was also suppressed with PARPi in the context of a severe acute respiratory syndrome coronavirus 2 (SARS-CoV-2) infection [63]. Therefore, reduced PARP-1 levels via inhibition, ubiquitin-mediated degradation, or knockdown can inhibit the inflammatory response.

## 3. PARP-1 and Cancer Progression

**Figure 1:**
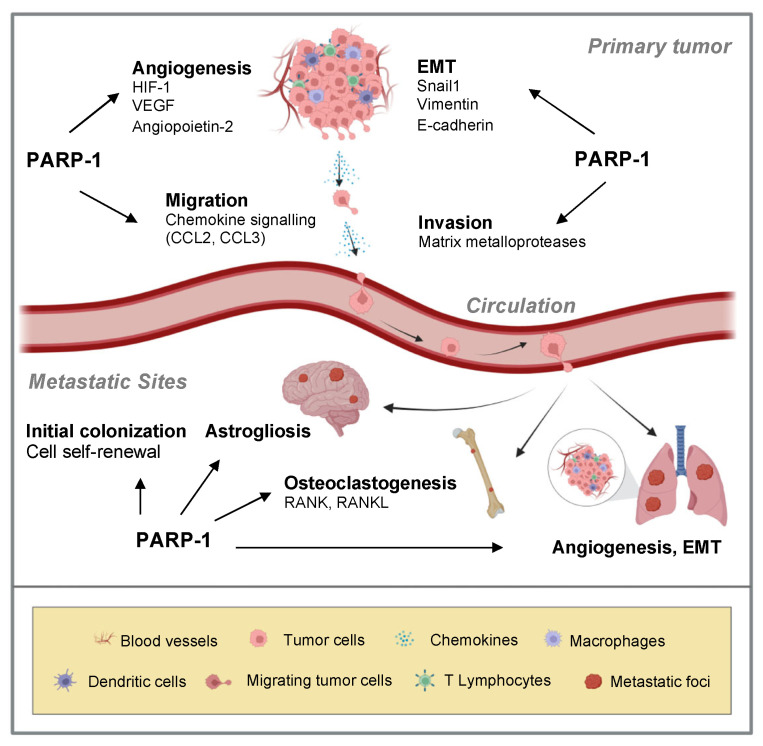
Role of PARP-1 in the metastatic cascade. During metastasis, PARP-1 promotes epithelial-to-mesenchymal transition (EMT) by regulating the expression of several proteins including vimentin, Snail1, and E-cadherin. PARP-1 induces the expression of matrix metalloproteases by activating the NF-κB pathway, facilitating the invasion of cancer cells through the extracellular matrix. In angiogenesis, PARP-1 regulates the transcriptional activity of HIF-1, leading to the expression of pro-angiogenic proteins. PARP-1 modulates cell migration by regulating the transcription of CCL2 through the transcriptional activity of NF-κB. PARP-1 also influences initial colonization, astrogliosis, osteoclastogenesis, angiogenesis, and EMT at distant metastatic sites, such as brain, bone, and lung.

The development of metastasis is a multistep process during which cancer cells undergo a cascade of events including primary tumor formation, localized invasion, angiogenesis and intravasation, transport through the circulation, arrest in microvessels of various organs, extravasation, formation of micrometastasis, and colonization with formation of macrometastasis distant sites [64,65]. The role of PARP-1 in the metastatic process is summarized in Figure 1.

### 3.1. PARP-1 and Tumor Microenvironment

PARP-1 activates different cell types including immune cells such as neutrophils, macrophages, and dendritic cells, and other cell types including endothelial cells and fibroblasts [47]. As such, PARP-1 modulates the tumor microenvironment by altering immune responses, chemokine expression, angiogenesis, and EMT.

#### 3.1.1. PARP-1 and Immunomodulatory Role

PARP-1 has been linked to immune modulation in multiple cancers, including breast, ovarian, and lung cancer [66]. In TNBC, the presence of an intact immune system, and particularly CD8+ T-cells, was critical for the efficacy of olaparib in vivo, prolonging survival by two-fold in comparison to treatment in an immunocompromised mouse [67]. PARPi induced an increase in CD3+ T cells, granzyme B-positive CD8+ T cells, and granzyme B-positive NK cells, which together suggest the activation of innate and adaptive immune responses. In *BRCA1/2*-deficient models in breast and ovarian cancer, PARPi increased cytosolic DNA and activated cGAS-STING (cyclic GMP-AMP synthase-stimulator of interferon genes) signalling, and upregulated pro-inflammatory chemokines such as CCL5 and CXCL10 [67,68,69]. Moreover, olaparib increased the levels and altered the phenotype of macrophages in the tumor microenvironment of TNBC [70]. PARP-1 was required for changes in the expression of CSF1R, which is associated with an immunosuppressive microenvironment. Indeed, combining PARPi with anti-CSF1R therapy reduced immunosuppressive macrophages and improved anti-tumor responses. Gene expression profiling has shown significant changes in the immune microenvironment in breast and ovarian cancers treated with olaparib. These include upregulation of transcripts of myeloid cells and macrophages, antigen presenting cells, chemokine- and cytokine-signalling cascades, Toll-like receptors, pro-inflammatory cytokine signalling, T-cell activation, and IFN-γ responses [68,70]. Taken together, these studies point toward an important role for PARP-1 in tumor immune signalling.

#### 3.1.2. PARP-1 and Chemokine Signalling

The impact of PARP-1 in chemokine signalling can be context-dependent. In ovarian cancer, PARPi activated stromal fibroblasts, which in turn increased CCL5 secretion [71]. In TNBC, PARP-1 knockdown with whole transcriptome analysis influenced cytokine signalling, with downregulation of *CCL2*, *CCL3*, and *CCL7* pathways. Knockdown of PARP-1 by siRNA led to an over 80% decrease in the abundance of *CCL2* mRNA in two different TNBC cell lines. With PAR levels as a measure of PARP-1 activity, a dose-dependent decrease in *CCL2* mRNA was also demonstrated upon PARP-1 inhibition, and *CCL2* increased with PARG inhibition [48]. However, in vivo, the stroma appears to be a major contributor to CCL2 activity. In oral cancer, stroma-derived CCL2 is a key player in recruiting myeloid-derived stem cells that express a receptor of CCL2, CCR2, from the bone marrow to the tumor microenvironment [72]. Furthermore, we previously demonstrated that the sequential combination of PARPi and carboplatin (with PARPi administered first), was associated with a downregulation of the *CCR5* signalling pathway in the mouse stroma, in which *CCL2* was a core-enriched gene [27].

Senescence-associated secretory phenotype (SASP) refers to the constellation of inflammatory, extracellular-modifying, and growth factors that are secreted by treatment-induced senescent cells [73]. While therapy-induced senescence can serve as an anti-tumor mechanism to inhibit cell proliferation and genomic instability, the persistence of therapy-induced senescence can create a pro-inflammatory microenvironment, promoting angiogenesis, EMT, cell migration, and metastasis [74]. Preclinical studies have reported several therapeutics such as PARPi, chemotherapy, and CDK4/6 inhibitors as inducers of senescence and SASP in ovarian, breast, and colon cancers [75,76,77]. However, the impact of PARPi upon senescence can also be context-dependent. In melanoma and breast cancer senescent cells, the SASP was driven by the PARP-1/ NF-κB signalling cascade [50]. Targeting PARP-1 was associated with the inability of NF-κB to relocate to the nucleus or stimulate NF-κB transcriptional activity, thereby inhibiting cell invasion and CCL2 secretion. Similar effects of PARPi were also observed when NF-κB transcriptional activity was upregulated in the context of chemotherapeutic drugs.

#### 3.1.3. PARP-1 and Angiogenesis

Angiogenesis, the process of new blood vessel formation from existing blood vessels, is an essential component of the metastatic cascade [78]. While inflammation and hypoxia are contexts where angiogenesis is promoted, pro-angiogenic factors, such as vascular endothelial growth factor (VEGF) or endothelial progenitor cells (EPCs) can be stimulated through autocrine, paracrine, or endocrine signalling mechanisms [79,80]. In particular, stromal fibroblasts in the primary tumor secrete SDF-1, which recruit EPCs from the bone marrow, promoting tumor angiogenesis [80]. One of the main regulators of angiogenesis in the context of hypoxia is hypoxia inducible factor (HIF)-1. Interestingly, PARP-1 is a transcriptional co-activator of HIF-1-dependent gene expression [81]. PARP-1 knockdown led to reduced *HIF1α* gene expression, thereby decreasing tumor vascularization [45]. Via direct interaction with *HIF-1*, the *PARP-1* and *HIF-1* complex can bind to the promoter of *NOTCH3*, which is involved in vascular invasiveness and metastasis [82]. PARPi can also inhibit VEGF-induced endothelial cell proliferation, migration, formation of tubule-like networks, and sprout formation [83,84]. PARP-1 knockdown in ovarian cancer cells reduced *VEGF* mRNA and protein secretion, hampering tubule formation of endothelial cells [85]. PARPi also significantly reduced the VEGF-induced activation of MAPK and AKT pathways (phosphorylation of ERK1/2, p38, and AKT) [86]. Furthermore, gene expression analysis of xenograft tumors of hepatocellular carcinoma treated with PARPi reduced expression of genes involved in angiogenesis (*HIF-2A*, *VEGFR1*, *ANG-2*) and genes regulated by *HIF-1α* [87]. Therefore, PARP-1 plays a key role in cancer angiogenesis.

#### 3.1.4. PARP-1 and EMT

EMT is a well-described process that helps explain tumor cell invasion and metastasis [88]. Here, cells lose their epithelial characteristics and close association with one another through the disassembly of cell–cell junctions and reorganization of the actin cytoskeleton, through the loss of E-cadherin expression. Cells acquire a mesenchymal phenotype associated with an increase in N-cadherin expression. Regulation of EMT occurs via transcription growth factor (TGF)-β, leading to the activation of Smad2/3, which mediates transcriptional regulation through three families of transcription factors: Snail (including Snail1, Snail2, or Slug), zing-finger E-box binding (ZEB), and basic helix-loop-helix (bHLH) [89]. Snail1/2 were shown to be important inducers of EMT [90].

However, the role of PARP-1 in EMT is not clear, with different studies reporting contradictory roles. PARP-1 activates Snail1 via gene transcription and PARylation. PARPi and PARP-1 knockdown led to the downregulation of Snail1 and increases in E-cadherin in melanoma cells [90]. Furthermore, PARP-1 interacts with Snail1 and the p65 subunit of NF-κB to activate the expression of fibronectin, a well-known mesenchymal marker [91,92]. In non-small cell lung carcinoma cells, PARP-1 knockdown resulted in a reversal of EMT with an increase in epithelial markers such as β-catenin, and a prominent decrease in mesenchymal markers such as vimentin [93]. PARP-1 was also shown to transcriptionally regulate vimentin by binding to the promoter of vimentin in lung cancer cells [94]. Moreover, PARPi prevented the development of EMT and partially reversed EMT in mammary gland cells [95].

Alternatively, studies have also shown that PARP-1 may downregulate EMT. PARP-1 can dissociate Smad complexes from DNA by PARylating Smad3/4, which reduces Smad responses and TGF-β induction of EMT [96]. In prostate cancer, functional inactivation of PARP-1 upregulates TGF-β levels and Smads, thereby inducing EMT and promoting tumorigenesis in vivo [97]. In a small set of BRCA^MUT^ patients (N = 4), treatment with the PARPi talazoparib was associated with an increase in a mesenchymal phenotype in two of the four patients [98]. Therefore, it is probable that tumor heterogeneity makes it difficult to understand the role of PARPi and EMT. In fact, several studies have emerged suggesting EMT as a critical determinant of PARPi resistance [99,100,101], warranting further investigation in patient samples.

#### 3.1.5. PARP-1 and Migration and Invasion

PARP-1 is involved in cell migration in several ways. PARP-1 overexpression stimulated cell migration 2- to 3-fold in non-small cell lung cancer cells [102]. PARPi suppressed endothelial cell migration, particularly when induced by VEGF or placental growth factor [83]. PARPi reduced the invasiveness and cell proliferation of ovarian cancer cells via PARP-1-mediated modulation of the NF-κB p65 subunit [103]. PARPi with olaparib also attenuated cell migration, invasion, and adhesion, with the inhibition of transcript levels of invasion markers *MMP2* and *MMP9*, while upregulating E-cadherins in oral and colon carcinoma cells [104,105]. By interacting with the NF-κB p65 subunit, PARP-1 was also shown to transcriptionally regulate *CCL2*. PARPi has resulted in the inhibition of cell migration in TNBC cells, with whole transcriptome analysis identifying marked downregulation of *CCL2* and *CCL3* pathways [48].

#### 3.1.6. PARP-1 and Hormone Receptors

The implication of PARP-1 has also been demonstrated in prostate cancer progression [106]. PARP-1 is highly PARylated in CRPC, in comparison with hormone-sensitive prostate cancer. PARP-1 is recruited to sites of androgen receptor (AR) transcriptional function, and PARP-1 enzymatic activity is required for AR-driven gene expression. Moreover, PARP-1 is required for AR function, in vivo tumor growth, and maintenance of castration resistance. PARP-1 also regulates ETS transcription factors, which may be under the control of AR activity in the context of prostate tumors with the *TMPRSS2-ERG* fusion [45].

### 3.2. PARP-1 and Metastasis

In a metastatic melanoma mouse model, targeting PARP-1 through stable expression of shRNA or PARPi improved survival [24]. PARPi inhibited distant lung metastasis by more than 80% with a reduction in tumor vessels at the primary tumor and metastatic site, in addition to a reduction in Snail1 expression and increase in E-cadherin expression at the metastatic site. Similarly, shRNA experiments targeting PARP-1 inhibited distant metastasis to the bone and brain in a lung adenocarcinoma mouse model [25]. Metastasis was promoted by PARP-1 by facilitating cell invasion, resistance to anoikis, extravasation, initial stages of metastatic colonization, and self-renewal. Interestingly, PARP-1 knockdown did not impact DNA damage or subcutaneous tumor growth in this model. The reduction in brain metastasis was associated with a reduction in astrogliosis. The metastasis-promoting effect of PARP-1 was upregulated by two transcription factors, S100A4 and CLDN7, thus independent of DNA repair.

The expression of PARP-1 has been evaluated in several cancer types. In melanoma patients, high PARP-1 expression was associated with worse melanoma-specific survival and overall survival (OS) for mucosal melanomas [107], and OS in late-stage metastatic melanomas [108]. In breast cancer patients who underwent surgery, patients with high PARP-1 expression demonstrated a four-fold higher risk of developing distant metastasis. It appeared plausible that PARP-1 may regulate the influence of TNF-α on breast cancer metastasis through the NF-κB signalling pathway [109]. Furthermore, in patients with lung adenocarcinoma, a high expression of PARP-1 was associated with poor OS and distant metastasis-free survival [25]. Similar prognostic implications of PARP-1 were also shown in soft-tissue sarcoma, colorectal cancer, and gastric cancer patients [110,111,112]. Therefore, there is a strong correlation between PARP-1 expression and metastasis, using patient samples in several cancer types.

## 4. PARP-2 Structure and Function in Cancer

In comparison to PARP-1, PARP-2 has a much lower molecular weight, at 66 kDa [113]. At the N-terminus, PARP-2 has nuclear and nucleolar localization signals and a WGR domain, which is the primary domain for DNA binding, as it lacks zinc-finger domains. The C-terminal domain of PARP-2 contains the catalytic domain which has high sequence and structural homology to PARP-1. As with PARP-1, HPF1 interacts with PARP-2 to stimulate the PARylation activity of PARP-2 [114]. Interestingly, along with differential targeting of DNA, PARP-1/2 have different PARylation activities toward protein substrates. A biochemical investigation of PARP-1/2 substrates identified 42 protein interactors of PARP-1 and 301 PARP-2 interactors [115]. Although PARP-2 contributes to only 5–15% of total PARylation activity [116], the differences in targeting mechanisms between PARP-1 and PARP-2 indicate that these PARylation events may lead to significant changes in subsequent cellular signalling.

PARP-2 plays an essential role in hematopoietic stem and progenitor cell (HSPC) survival in steady-state or stress response conditions [117]. PARP-2-deficient mice were associated with an increase in HSPC death, and in response to radiation, PARP-2 deficiency, and not PARP-1 deficiency, resulted in bone marrow failure. In TNBC, selective degradation of PARP-2 using a proteolysis-targeting chimera (PROTAC) showed significant anti-tumour effects in both cell lines and xenograft models [118]. In breast cancer mouse models, PARP-2 deficiency did not affect tumor growth rate, but was associated with delayed tumor onset and a significant reduction in lung metastasis [119]. However, one study reported that PARP-2 depletion, and not PARP-1 depletion, was associated with an increase in bone metastasis [119]. PARP-2 expression has also been associated with prostate cancer progression [113]. Selective targeting of PARP-2 inhibited AR-positive prostate cancer cell growth and tumor growth in vivo. Therefore, while there is an increasing interest in elucidating the role of PARP-2, more studies are required to better understand its role in the microenvironment and distant metastasis.

## 5. PARP Inhibitors

### 5.1. Mechanism of Action

PARPi are known to function by inhibiting DNA repair and inducing DNA damage [120]. The most well-described mechanism is that of synthetic lethality in BRCA^MUT^ cells [120,121]. PARPi block PARylation by competitively binding to the NAD^+^ site of PARP-1 and PARP-2. PARPi lead to the accumulation of unrepaired single-strand DNA breaks that are converted to double-strand DNA breaks. In *BRCA1/2*-proficient cells, active BRCA1/2 will repair those damages leading to cell survival, while in BRCA^MUT^ cells, double-strand DNA breaks accumulate, resulting in cell death. However, PARPi have also been shown to induce DNA damage by the formation of PARP-DNA complexes. Here, PARP-1/2 bind the 5′-deoxyribose phosphate group-containing DNA ends to form toxic PARP-DNA complexes that strongly block DNA replication, thereby inducing double-strand breaks with bulky PARP proteins at one strand of 5′ DNA ends [122]. The PARP-DNA trapping mechanism helps explain the efficacy of PARPi in *BRCA1/2* wild-type cells, as trapped PARP-DNA complexes require alterations in multiple DNA repair pathways.

### 5.2. Different PARPi

First generation PARPi target both PARP-1 and PARP-2, and include veliparib, olaparib, rucaparib, niraparib, and talazoparib [123]. The different PARPi have similar abilities to inhibit the enzymatic activity of PARP-1/2 but differ in their ability to trap PARP-1 onto the DNA strand. As such, PARPi can be ranked from lowest to highest potency based on their respective PARP-DNA trapping ability: veliparib, olaparib, rucaparib, niraparib, and talazoparib [121]. More recently, selective PARPi that target only PARP-1 are being developed and have shown promising results [124,125]. For example, saruparib demonstrated high selectivity for PARP-1, with anti-tumor efficacy in BRCA^MUT^ mouse models of breast, colon, and pancreatic cancer, and reduced hematological toxicity [124]. Another selective and potent PARP-1 inhibitor, AZD9574, revealed efficacy in breast and ovarian cancer cells [125]. Of note, four of the first-generation PARPi, namely olaparib, rucaparib, niraparib, and talazoparib have been approved by the U.S. FDA, and three of these PARPi, including olaparib, niraparib, and talazoparib, were approved by Health Canada [12,126].

### 5.3. Preclinical Studies of PARPi in Combination to Inhibit Distant Metastasis

Several PARPi combination approaches have been used to demonstrate strong inhibition of distant metastasis in pre-clinical mouse models. One such approach targets CXCR4, which we and others have shown to be a strong prognostic biomarker and important therapeutic target in breast cancer [127,128]. In a BRCA^WT^ TNBC model, the CXCR4 inhibitor AMD3100 suppressed the development of distant metastasis when combined with olaparib [129]. PARP-1 expression was shown to be regulated by CXCR4 and the combination of AMD3100 and olaparib demonstrated inhibition of cell migration. Moreover, this combination strategy enhanced DNA damage and apoptosis. Another combination approach targets a transcription factor, KLF5 (Kruppel-like factor 5), which promotes ovarian cancer growth and metastasis [130]. Knockdown of KLF5 downregulated the homologous recombination repair pathway including RAD51 expression. Targeting KFL5 with a histone deacetylase (HDAC) inhibitor, suberoylanilide hydroxamic acid (SAHA), in combination with olaparib, significantly inhibited abdominal metastasis in comparison to single-agent therapy. In the context of melanoma, PARPi inhibited lung metastasis, which was associated with a reduced number of tumor vessels and expression of EMT markers at the metastatic site [24]. In combination, PARPi plus MAPK inhibition demonstrated synergy in melanoma due to a DNA repair-independent mechanism [131]. This combination induced cell death via autophagy and attenuated distant metastasis in patient-derived xenograft models.

We previously investigated the combination of talazoparib and carboplatin using different sequencing strategies in TNBC [27]. We demonstrated that talazoparib and carboplatin were synergistic in 92% of TNBC cell lines (12/13) using a concomitant dosing strategy. We showed that lower concentrations of talazoparib and carboplatin were required in combination to induce DNA damage. We evaluated different sequencing strategies of PARPi with carboplatin, i.e., PARPi-first, carboplatin-first, and concomitant administration, since sequenced PARPi combination approaches were possibly associated with decreased toxicity and improved efficacy [132,133]. We showed that all combination approaches inhibited cell proliferation and tumor growth in TNBC cell lines and orthotopic xenograft models in a comparable manner. However, the sequential PARPi-first combination demonstrated the greatest inhibition of cell migration and invasion and was the most effective strategy in inhibiting lung metastasis, by 56%, which was not observed with the concomitant combination. Whole transcriptome analysis of the metastatic lung tissue demonstrated that the PARPi-first sequential combination group downregulated DNA repair and replication pathways. However, the concomitant combination approach was associated with a proinflammatory phenotype with an upregulation of VEGF and chemokine signalling pathways. While there could be several explanations, it is plausible that the concomitant approach induces extensive DNA damage, thereby promoting the proinflammatory response and possibly tumor progression [134].

It is also important to understand the site-specificity of PARPi in two particular contexts—bone and brain metastasis. While an initial study reported an induction of bone metastasis with PARPi, being mainly mediated by PARP-2 in the myeloid lineage and less by PARP-2 in cancer cells, this study administered PARPi first for one week prior to the injection of cancer cells [119]. Two subsequent studies reported contrasting results [26,104]. First, in the context of oral squamous cell carcinoma, olaparib markedly suppressed tumor invasion to the bone, in comparison to the control group which was associated with destruction of the zygoma arch, ramus, mandibular angle, and external auricular canal [104]. Olaparib reducted osteoclastogenesis, evidenced by decreased expression in RANK, RANKL, and EMT markers, with a reduction in Snail1 and increase in E-cadherin expression. Osteoclast activity can also be inhibited by zoledronate [26]. Interestingly, the combination of zoledronate and olaparib decreased glutamine levels in the bone microenvironment, thereby demonstrating a synergistic effect in inhibiting breast cancer bone metastasis in vivo.

PARPi have also been studied in the context of brain metastasis [125,135]. Pamiparib, a PARPi which targets PARP-1 and PARP-2, demonstrated higher potency than olaparib [135]. In comparison to olaparib, talazoparib, and niraparib, pamiparib demonstrated the highest drug exposure in the brain. In a xenograft model of small cell lung cancer brain metastasis, the combination of pamiparib and temozolomide prolonged survival in comparison to temozolomide alone. The combination also improved survival in a glioma model, suggesting that pamiparib has a strong penetration across the blood–brain barrier. More recently, a PARP-1 selective inhibitor, AZD9574, was evaluated in primary and metastatic brain tumors [125]. AZD9574 demonstrated higher permeability across the blood–brain barrier than pamiparib. In a *BRCA1*-mutant intracranial metastatic breast cancer model, AZD9574 led to significant tumor regression and improvement in survival, in comparison to olaparib. Therefore, with the advent of next-generation PARPi, there may be a greater utility in the context of brain metastasis.

## 6. Recent Clinical Advances of PARPi

### 6.1. PARPi in Breast Cancer

There are several clinical trials that have demonstrated the role of PARPi at various stages of the metastatic process. Since we have previously reviewed the clinical utility of PARPi as a monotherapy and in combination in breast cancer [13], here, we focus on the more recent advancements regarding PARPi in the context of metastasis. The first phase III randomized controlled trial that compared olaparib versus single-agent treatment of physician’s choice in 302 patients with gBRCA^MUT^ HER2-negative metastatic breast cancer was the OlympiAD trial [14]. Initial results demonstrated an improvement in progression-free survival (PFS) in patients receiving olaparib, (7.0 vs. 4.2 months; hazard ratio (HR) for disease progression or death 0.58; 95% CI, 0.43–0.80; *p* < 0.001) [14]. Interestingly, with an extended follow-up, patients treated with olaparib showed a possible improvement in OS amongst all patients (19.3 versus 17.1 months, HR 0.89, 95% CI, 0.67–1.18), which was more pronounced in the first-line setting (without prior chemotherapy) (22.6 versus 14.7 months, HR 0.55, 95% CI, 0.33–0.95) [136]. Therefore, these results are suggestive of the greater benefit of olaparib if administered as an earlier line of treatment for gBRCA^MUT^ HER2-negative metastatic breast cancer patients.

The benefit for olaparib was also demonstrated in the adjuvant setting for breast cancer patients. OlympiA, a phase III clinical trial, comprised 1836 early breast cancer patients carrying a pathogenic *BRCA1/2* variant with TNBC or hormone receptor-positive HER2-negative breast cancer and compared 1 year of olaparib versus a placebo [137]. Patients treated with olaparib showed a statistically significant improvement in four-year OS (89.8% versus 86.4%; 95% CI, −0.1%–6.8%) and distant disease-free survival (86.5% versus 79.1%; 95% CI, 3.6–11.3%) [15]. This study led to the approval of olaparib by the FDA in the adjuvant setting of gBRCA^MUT^ breast cancer patients [138], highlighting the importance of genetic testing in the surgical and medical treatment decision pathway in HER2-negative breast cancer [7]. While it is well known that ~15% of TNBCs are associated with *BRCA1* mutations [13], the frequency of gBRCA^MUT^ is up to 8% of all hormone receptor-positive breast cancers, and about 40% of breast cancers with low hormone receptor expression [139]. However, since TNBCs constitute about 15% of all breast cancers, while hormone receptor-positive breast cancers comprise about 70% of all breast cancers, there is actually a greater number of hormone receptor-positive breast cancer patients who are gBRCA^MUT^. Moreover, from a biological perspective, the significance of olaparib in the adjuvant setting in improving distant disease-free survival and OS is indicative that PARPi can inhibit micrometastasis.

Recent studies have also demonstrated the efficacy of PARPi either as a monotherapy or in combination in TNBC patients [13]. The PETREMAC trial demonstrated an objective response of 56% with single-agent olaparib in an unselected cohort of TNBC patients without prior exposure to chemotherapy [140]. Similarly, 58% of patients treated with rucaparib were associated with greater than 75% decline in circulating tumor DNA in the neoadjuvant setting [141]. Interestingly, in a recent phase II clinical trial of patients with advanced TNBC and prior platinum therapy, olaparib as monotherapy was associated with a median overall survival of 21.7 months, in which only 1 of 23 patients had a known deleterious mutation in *BRCA1/2* [142]. Furthermore, the combination of cisplatin and veliparib was evaluated in metastatic TNBC patients. The only subgroup that demonstrated a statistically significant difference in PFS between the combination and cisplatin alone were patients with HRD (BRCA-like) (5.9 versus 4.2 months, HR 0.57; 95% CI, 0.37–0.88, *p* = 0.010) [143]. Therefore, these results are suggestive that there is likely a larger population of TNBC patients, outside of gBRCA^MUT^ patients, who can benefit from PARPi.

### 6.2. Role of PARPi Combination Strategies

PARPi have been evaluated in several trials in patients with metastatic CRPC [144]. Here, the patient population that is eligible for PARPi are those with either gBRCA^MUT^ or mutations in homologous recombination repair genes. Earlier studies evaluated PARPi as a monotherapy, either PARPi in the context of BRCA^MUT^ patients who progressed after AR therapy and chemotherapy, or patients with any HRD gene mutations post-AR therapy [17]. In comparison to AR therapy, olaparib demonstrated an improvement in OS (HR 0.69; 95% CI, 0.5–0.97; *p* = 0.02) [8]. More recently, trials have evaluated the use of PARPi in combination as a first-line approach for metastatic CRPC. The combination of olaparib and abiraterone, an androgen biosynthesis inhibitor [145] plus prednisone demonstrated an improvement in radiographic PFS particularly in BRCA^MUT^ patients (HR 0.24; 95% CI, 0.12–0.45) [146]. Furthermore, talazoparib in combination with a nonsteroidal AR inhibitor, enzulatamide, demonstrated an improvement in radiographic PFS in comparison to the placebo plus enzalutamide (HR 0.45; 95% CI, 0.33–0.61, *p* < 0.0001) in a phase III randomized controlled trial [147]. Therefore, the combination of PARPi and anti-hormonal therapy plays an important role in slowing the metastatic progression in prostate cancer.

In ovarian cancer, PARPi were initially approved as a monotherapy for BRCA^MUT^ patients with recurrent disease, but there is an increasing use of PARPi as a maintenance therapy in the primary setting, after completion of initial therapy upon diagnosis [18]. The benefit of PARPi as a monotherapy is well established in the primary setting due to improvements in OS and PFS, but we will focus our discussion on first-line maintenance combination therapy. The first study to demonstrate the efficacy of the combination of a PARPi and angiogenesis inhibitor was the phase III PAOLA-1 trial [148]. Here, olaparib was combined with bevacizumab, a monoclonal antibody which targets VEGF-A, and demonstrated an improvement in PFS in comparison to bevacizumab alone (22.1 versus 16.6 months; HR 0.59; 95% CI 0.49–0.72; *p* < 0.001) amongst all patients, regardless of *BRCA1/2* mutation status. Subgroup analysis demonstrated a more pronounced benefit amongst patients with HRD tumors, defined by a commercially available test (MyChoice^®^CDx, which combines loss of heterozygosity, telomeric allelic imbalance, and large-scale transitions). At 5-year follow-up, the combination demonstrated an improvement in OS (HR 0.62; 95% CI 0.45–0.85) [149]. A systematic review and meta-analysis of the combination of angiogenesis inhibitors and PARPi also showed an improvement in PFS, in comparison to either PARPi or anti-angiogenesis agents (HR 0.62; 95% CI, 0.52–0.73) [150]. Despite the approval of this combination by the FDA, the strategic use of this combination has been pointed out to be challenging in the clinic [151,152].

With a strong pre-clinical rationale, several clinical trials have evaluated the combination of PARPi and immune checkpoint inhibitors. We will discuss four of the more recent trials, as we and others have reviewed previous studies in breast cancer [13,20]. In a phase I/IIb non-randomized study, the JAVELIN PARP Medley trial evaluated the combination of talazoparib and avelumab in advanced solid tumors [153]. The objective response rate was the highest, at 63% amongst *BRCA1/2*-altered platinum-sensitive ovarian cancer patients, and a prolonged duration of response of 11.1 months identified for TNBC patients. In a pan-cancer non-randomized phase IIb trial including patients with *BRCA1/2*-altered tumors (JAVELIN BRCA/ATM), the combination of talazoparib and avelumab demonstrated an objective response rate of 26.4%, not achieving the pre-specified rate of 40% [153]. DORA is a phase II non-comparative study of olaparib and olaparib plus durvalumab in platinum pre-treated advanced TNBC patients [142]. For patients treated with the combination, the median PFS was 6.1 months (95% CI, 3.7–10.1), which was longer than historical controls (*p* < 0.0001). In the first randomized controlled trial that compared olaparib with atezolizumab in gBRCA^MUT^ advanced breast cancer patients, the addition of atezolizumab did not improve PFS in comparison with olaparib alone, but the combination was well tolerated [154]. Therefore, while the advantage of combining PARPi with immune checkpoint inhibitors appears to be underwhelming, more studies are required to better understand patient selection and sequencing of therapies. Of note, in the context of gBRCA^MUT^ TNBC patients with residual disease post-neoadjuvant chemoimmunotherapy, while clinicians tend to combine PARPi with pembrolizumab in the adjuvant setting, clinical trials have yet to demonstrate improved efficacy with such a combination.

### 6.3. Impact of PARP Inhibitors and Site-Specific Distant Metastasis

PARPi may have differential efficacy at specific sites of distant metastasis, suggesting the differential role of the microenvironment in influencing therapeutic efficacy. In the OlympiA trial, the frequency of distant metastasis that patients developed in follow-up can be numerically compared. Olaparib (N = 921), in comparison to placebo (N = 915), was associated with a lower frequency of distant metastasis at the following sites: CNS recurrence (2.4% (N = 22) versus 3.9% (N = 36)), bone (0.5% (N = 5) versus 1.5% (N = 14)), distant lymph nodes (0.5% (N = 5) versus 1.0% (N = 9)), and lung (1.7% (N = 16) versus 3.7% (N = 34)). However, the frequencies of liver metastasis and pleural effusions was more comparable between the two groups (2.2% (N = 20) versus 2.5% (N = 23) and 0.3% (N = 3) versus 0.4% (N = 4), respectively). Several case reports have identified a role for PARPi in patients with CNS metastasis. For *BRCA1/2*-related and BRCA^WT^ patients with ovarian cancer brain metastasis treated with olaparib or niraparib as maintenance therapy, PFS ranged from 9 months to 4 years [155,156]. A *BRCA2*-mutant breast cancer patient with leptomeningeal carcinomatosis demonstrated a complete clinical and radiologic response to olaparib after 19 months of treatment [157]. Therefore, while there are interesting preliminary data regarding the impact of PARPi in inhibiting the development and progression of distant metastasis, further studies are required to better understand site-specificity.

### 6.4. Next-Generation PARP Inhibitors

Future clinical trials with PARPi will be evaluating the selective inhibition of PARP-1 instead of the first-generation PARPi, which target both PARP-1 and PARP-2 [158,159]. The overarching aim with a selective PARP-1 inhibitor is to overcome the adverse effects related to PARP-2 inhibition, namely anemia, neutropenia, and thrombocytopenia [160,161]. Saruparib, a selective PARP-1 inhibitor, was tested in patients with advanced solid tumors with mutations in *BRCA1/2*, *PALB2*, or *RAD51* in the PETRA trial [162]. Preliminary results of breast cancer cohort identified the recommended a phase II dose of 60 mg po daily, with a median PFS of 9.1 months (80% CI, 5.7–9.3 months), and a favorable safety profile, suggesting the potential for longer treatment durations and improved efficacy. Safety has also been demonstrated for another PARP-1 selective inhibitor, namely HRS-1167 [163], and a phase I trial is ongoing for an additional PARP-1 selective inhibitor, IMP1734 [164], all in advanced solid tumors, suggesting the clinical potential for this targeted agent.

## 7. Conclusions

While the implication of PARP-1/2 in DNA repair is undisputed, the multifaceted role of PARP-1 cannot be overlooked. PARP-1 has been shown to regulate expression of genes such as *CCL2*, *SDF-1*, *HIF-1*, *Snail1*, and *Vimentin*, which are key players at various stages of the metastatic process, including chemokine signalling, angiogenesis, and EMT. Recent studies have evaluated PARPi combination approaches in vivo to better understand the role of the tumor and microenvironment in the inhibition of distant metastasis. The combination of PARPi and a CXCR4 inhibitor increased DNA damage and apoptosis. HDAC inhibition was associated with a downregulation of homologous recombination repair pathways, thereby mediating sensitivity in olaparib-resistant cells. Downregulation of DNA repair pathways was also observed with the sequential combination of PARPi and carboplatin. PARPi combinations have also demonstrated efficacy via mechanisms independent of DNA repair. The sequential PARPi combination with carboplatin downregulated chemokine signalling pathways in the stroma of the metastatic site. The combination of PARPi and MAPK inhibition induced cell death by autophagy. Furthermore, olaparib plus zoledronate synergistically inhibited bone metastasis by blocking osteoclast activity and decreasing glutamine levels.

Clinically, PARPi were initially approved in the metastatic or recurrent setting as a monotherapy for BRCA^MUT^ patients. While current PARPi have shown promise in improving outcomes for patients with brain metastasis, further investigation is needed with newer PARPi and PARP-1 selective inhibitors that possess an enhanced ability to cross the blood–brain barrier. However, in breast and ovarian cancers, the utility of PARPi has shifted from advanced cancers to the primary setting, either as adjuvant or maintenance therapy as monotherapy. Combination therapy with an angiogenesis inhibitor has also shown benefits for HRD patients in the primary setting of ovarian cancer patients. Therefore, PARPi have demonstrated efficacy in both slowing the progression of metastasis and impeding the development of distant metastasis.

With the expanding utility of PARPi in the clinic, it has become increasingly important to understand the factors that influence PARPi response and resistance. Although studies have focused on the genetic determinants of PARPi response at the primary tumor, a more comprehensive evaluation of the tumor, microenvironment, and metastatic process will uncover critical vulnerabilities and biomarkers, leading to new combination approaches and improved patient selection that can benefit from PARPi. Continued research and clinical trials will be crucial to unlock the full potential of PARPi, to inhibit the development of distant metastasis, and to significantly improve the survival of patients with difficult-to-treat cancers.

## Data Availability

Not Applicable.
